# Crystal structure of (*Z*)-3-benz­yloxy-6-[(2-hy­droxy-5-methyl­anilino)methyl­idene]cyclo­hexa-2,4-dien-1-one

**DOI:** 10.1107/S1600536814016936

**Published:** 2014-08-01

**Authors:** Nadir Ghichi, Ali Benosmane, Ali Benboudiaf, Hocine Merazig

**Affiliations:** a Unité de Recherche de Chimie de l’Environnement et Moléculaire Structurale (CHEMS), Faculté des Sciences Exactes, Département de Chimie, Université Constantine 1, Algeria

**Keywords:** crystal structure, Schiff base, azomethines

## Abstract

In the title Schiff base compound, C_21_H_19_NO_3_, the conformation about the C=C bond is *Z*. The N—H group and carbonyl O atom form an intra­molecular N—H⋯O hydrogen bond with an *S*(6) ring motif. The benz­yloxy ring and the 2-hy­droxy-5-methyl­phenyl ring are inclined to the central six-membered ring by 13.68 (9) and 9.13 (8)°, respectively, and to one another by 21.95 (9)°. In the crystal, mol­ecules are linked by O—H⋯O hydrogen bonds, forming helical chains along [010].

## Related literature   

For some general background on Schiff bases and their various biological activities, see: Arora *et al.* (2002 ▶); El-Masry *et al.* (2000 ▶); Jarrahpour & Khalili (2006 ▶); More *et al.* (2001 ▶); Phatak *et al.* (2000 ▶). For related structures, see: Akkurt *et al.* (2005 ▶, 2008 ▶). For pharmaceutical and industrial applications of azomethines, see: Prakash & Adhikari (2011 ▶). For the effect of hydro­philicity on drug properties, see: Lin & Lu (1997 ▶).
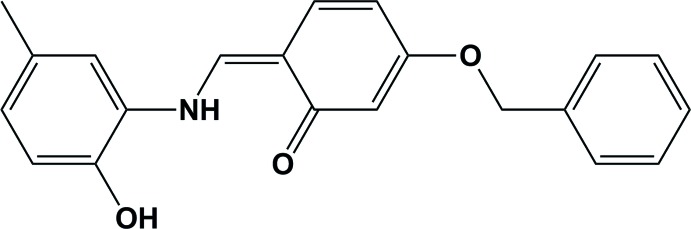


## Experimental   

### Crystal data  

C_21_H_19_NO_3_
*M* *_r_* = 333.37
Monoclinic, 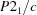

*a* = 12.594 (5) Å
*b* = 9.303 (5) Å
*c* = 14.997 (5) Å
β = 96.402 (5)°
*V* = 1746.1 (13) Å^3^
*Z* = 4
Mo *K*α radiation
μ = 0.09 mm^−1^
*T* = 293 K
0.03 × 0.02 × 0.01 mm

### Data collection  

Bruker APEXII CCD diffractometer
19603 measured reflections
5148 independent reflections
2672 reflections with *I* > 2σ(*I*)
*R*_int_ = 0.037

### Refinement  

*R*[*F* ^2^ > 2σ(*F* ^2^)] = 0.050
*wR*(*F* ^2^) = 0.158
*S* = 1.01
5148 reflections
238 parameters
H atoms treated by a mixture of independent and constrained refinement
Δρ_max_ = 0.22 e Å^−3^
Δρ_min_ = −0.16 e Å^−3^

### 

Data collection: *APEX2* (Bruker, 2006 ▶); cell refinement: *SAINT* (Bruker, 2006 ▶); data reduction: *SAINT*; program(s) used to solve structure: *SHELXS97* (Sheldrick, 2008 ▶); program(s) used to refine structure: *SHELXL97* (Sheldrick, 2008 ▶); molecular graphics: *ORTEP-3 for Windows* (Farrugia, 2012 ▶) and *Mercury* (Macrae *et al.*, 2008 ▶); software used to prepare material for publication: *WinGX* (Farrugia, 2012 ▶).

## Supplementary Material

Crystal structure: contains datablock(s) global, I. DOI: 10.1107/S1600536814016936/su2761sup1.cif

Structure factors: contains datablock(s) I. DOI: 10.1107/S1600536814016936/su2761Isup2.hkl

Supporting information file. DOI: 10.1107/S1600536814016936/su2761Isup3.cml

**Fig. 1.**
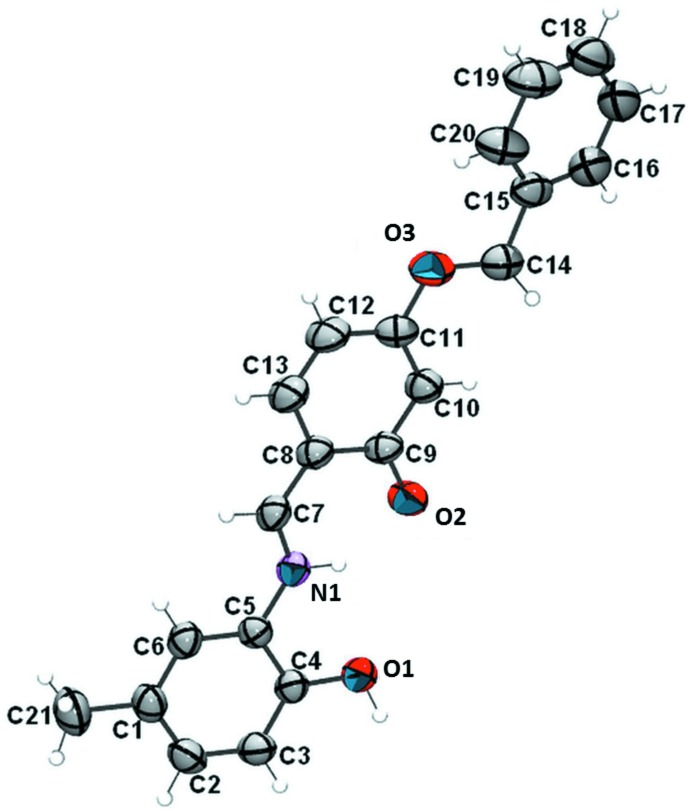
View of the molecular structure of the title molecule, with atom labelling. Displacement ellipsoids are drawn at the 50% probability level.

**Fig. 2.**
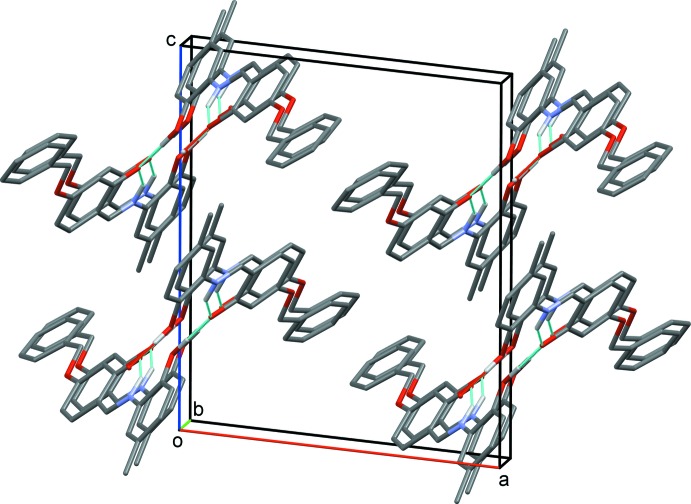
Partial view along the b axis of the crystal packing of the title compound, showing the hydrogen bonds as dashed lines (see Table 1 for details).

. DOI: 10.1107/S1600536814016936/su2761fig1.tif
View of the mol­ecular structure of the title mol­ecule, with atom labelling. Displacement ellipsoids are drawn at the 50% probability level.

b . DOI: 10.1107/S1600536814016936/su2761fig2.tif
Partial view along the *b* axis of the crystal packing of the title compound, showing the hydrogen bonds as dashed lines (see Table 1 for details).

CCDC reference: 1015522

Additional supporting information:  crystallographic information; 3D view; checkCIF report

## Figures and Tables

**Table 1:** Hydrogen-bond geometry (Å, °)

*D*—H⋯*A*	*D*—H	H⋯*A*	*D*⋯*A*	*D*—H⋯*A*
N1—H1*n*⋯O2	0.94 (2)	1.83 (2)	2.609 (2)	138.7 (16)
O1—H1*o*⋯O2^i^	0.96 (2)	1.63 (2)	2.590 (2)	176.1 (17)

Symmetry code: (i) 
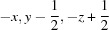
.

## supplementary crystallographic information

### S1. Experimental

A mixture of 2-amino-4-methylphenol (1 mmol) and 4-(benzyloxy)-2-hydroxybenzaldehyde (1 mmol) was heated to form a clear solution. To this a few drops of conc. HCL was added as a catalyst and the mixture was refluxed for 12 h. After cooling the solution to 80°C it was stirred for 45 min the a precipitate formed. It was filtered off and washed with ice cold ethyl acetate to give the pure title Schiff base compound as an orange solid (yield 35%). This crude product was dissolved in ethyl acetate and two spoons of activated charcoal were added. The mixture was filtered over celiteµ000174 and the product was crystallized from ethyl acetate. The compound was very difficult to crystalize and only after several attempts over a period of four months were crystals suitable for X-ray diffraction analysis finally obtained.

### S2. Refinement

The OH and NH H atoms, and the methine H atom were located in a difference Fourier map and freely refined. The C-bound H atoms were fixed geometrically and treated as riding atoms: C—H = 0.93 Å (aromatic) and 0.97 Å (methylene) with U_iso_(H) = 1.2U_eq_(C).

### Crystal data

**Table:**

C_21_H_19_NO_3_	*Z* = 4
*M**_r_* = 333.37	*F*(000) = 704
Monoclinic, *P*2_1_/*c*	*D*_x_ = 1.268 Mg m^−^^3^
Hall symbol: -P 2ybc	Mo *K*α radiation, λ = 0.71073 Å
*a* = 12.594 (5) Å	µ = 0.09 mm^−^^1^
*b* = 9.303 (5) Å	*T* = 293 K
*c* = 14.997 (5) Å	Block, orange
β = 96.402 (5)°	0.03 × 0.02 × 0.01 mm
*V* = 1746.1 (13) Å^3^	

### Data collection

**Table:**

Bruker APEXII CCD diffractometer	2672 reflections with *I* > 2σ(*I*)
Radiation source: fine-focus sealed tube	*R*_int_ = 0.037
Graphite monochromator	θ_max_ = 30.2°, θ_min_ = 2.6°
phi and ω scans	*h* = −17→17
19603 measured reflections	*k* = −13→13
5148 independent reflections	*l* = −21→20

### Refinement

**Table:**

Refinement on *F*^2^	Primary atom site location: structure-invariant direct methods
Least-squares matrix: full	Secondary atom site location: difference Fourier map
*R*[*F*^2^ > 2σ(*F*^2^)] = 0.050	Hydrogen site location: inferred from neighbouring sites
*wR*(*F*^2^) = 0.158	H atoms treated by a mixture of independent and constrained refinement
*S* = 1.01	*w* = 1/[σ^2^(*F*_o_^2^) + (0.0718*P*)^2^ + 0.1066*P*] where *P* = (*F*_o_^2^ + 2*F*_c_^2^)/3
5148 reflections	(Δ/σ)_max_ < 0.001
238 parameters	Δρ_max_ = 0.22 e Å^−^^3^
0 restraints	Δρ_min_ = −0.16 e Å^−^^3^

### Special details

**Table:**

Geometry. Bond distances, angles *etc*. have been calculated using the rounded fractional coordinates. All su's are estimated from the variances of the (full) variance-covariance matrix. The cell e.s.d.'s are taken into account in the estimation of distances, angles and torsion angles
Refinement. Refinement of *F*^2^ against ALL reflections. The weighted *R*-factor *wR* and goodness of fit *S* are based on *F*^2^, conventional *R*-factors *R* are based on *F*, with *F* set to zero for negative *F*^2^. The threshold expression of *F*^2^ > σ(*F*^2^) is used only for calculating *R*-factors(gt) *etc*. and is not relevant to the choice of reflections for refinement. *R*-factors based on *F*^2^ are statistically about twice as large as those based on *F*, and *R*- factors based on ALL data will be even larger.

### Fractional atomic coordinates and isotropic or equivalent isotropic displacement parameters (Å^2^)

**Table:**

	*x*	*y*	*z*	*U*_iso_*/*U*_eq_	
O1	0.00597 (9)	0.00730 (11)	0.29239 (8)	0.0607 (4)	
O2	0.11904 (9)	0.35839 (11)	0.32139 (8)	0.0632 (4)	
O3	0.35952 (9)	0.74624 (12)	0.39999 (9)	0.0686 (4)	
N1	0.14153 (10)	0.12579 (13)	0.41887 (9)	0.0466 (4)	
C1	0.10315 (13)	−0.23275 (17)	0.52145 (11)	0.0543 (5)	
C2	0.03317 (14)	−0.29008 (17)	0.45329 (12)	0.0599 (6)	
C3	−0.00117 (13)	−0.21414 (16)	0.37654 (11)	0.0557 (5)	
C4	0.03510 (12)	−0.07513 (15)	0.36568 (10)	0.0464 (5)	
C5	0.10653 (11)	−0.01575 (15)	0.43336 (10)	0.0435 (4)	
C6	0.13925 (12)	−0.09409 (16)	0.51009 (10)	0.0502 (5)	
C7	0.21681 (12)	0.19787 (17)	0.46691 (11)	0.0500 (5)	
C8	0.24727 (11)	0.33802 (16)	0.44826 (10)	0.0488 (5)	
C9	0.19690 (11)	0.41507 (16)	0.37232 (11)	0.0488 (5)	
C10	0.23632 (12)	0.55360 (16)	0.35497 (11)	0.0535 (5)	
C11	0.31795 (12)	0.61279 (17)	0.41101 (11)	0.0549 (6)	
C12	0.36589 (14)	0.53810 (18)	0.48665 (13)	0.0654 (6)	
C13	0.33182 (13)	0.40505 (18)	0.50384 (12)	0.0626 (6)	
C14	0.31877 (13)	0.82640 (18)	0.32322 (12)	0.0600 (6)	
C15	0.37303 (14)	0.96959 (18)	0.32326 (12)	0.0597 (6)	
C16	0.32694 (18)	1.0777 (2)	0.26853 (13)	0.0769 (8)	
C17	0.3757 (2)	1.2105 (2)	0.26580 (14)	0.0893 (9)	
C18	0.4695 (2)	1.2378 (2)	0.31800 (16)	0.0918 (10)	
C19	0.51594 (18)	1.1327 (2)	0.37257 (18)	0.0933 (9)	
C20	0.46785 (16)	0.9979 (2)	0.37561 (16)	0.0781 (8)	
C21	0.13941 (16)	−0.3163 (2)	0.60556 (13)	0.0792 (8)	
H1o	−0.0411 (17)	−0.044 (2)	0.2490 (16)	0.101 (7)*	
H2	0.00830	−0.38330	0.45937	0.0719*	
H1n	0.1091 (14)	0.1794 (19)	0.3701 (13)	0.075 (6)*	
H3	−0.04862	−0.25606	0.33208	0.0668*	
H6	0.18647	−0.05266	0.55491	0.0603*	
H7	0.2525 (13)	0.1493 (17)	0.5192 (12)	0.065 (5)*	
H10	0.20650	0.60482	0.30510	0.0642*	
H12	0.42068	0.58043	0.52442	0.0785*	
H13	0.36443	0.35558	0.55341	0.0750*	
H14A	0.24242	0.84004	0.32335	0.0720*	
H14B	0.33057	0.77387	0.26933	0.0720*	
H16	0.26261	1.06082	0.23328	0.0922*	
H17	0.34437	1.28183	0.22815	0.1070*	
H18	0.50154	1.32764	0.31634	0.1101*	
H19	0.57997	1.15094	0.40792	0.1119*	
H20	0.49987	0.92686	0.41309	0.0937*	
H21A	0.10696	−0.40980	0.60202	0.1186*	
H21B	0.11862	−0.26601	0.65674	0.1186*	
H21C	0.21574	−0.32628	0.61143	0.1186*	

### Atomic displacement parameters (Å^2^)

**Table:**

	*U*^11^	*U*^22^	*U*^33^	*U*^12^	*U*^13^	*U*^23^
O1	0.0764 (8)	0.0485 (6)	0.0514 (7)	−0.0042 (5)	−0.0186 (6)	0.0026 (5)
O2	0.0626 (7)	0.0505 (6)	0.0687 (8)	−0.0121 (5)	−0.0277 (6)	0.0067 (5)
O3	0.0682 (7)	0.0572 (7)	0.0771 (9)	−0.0194 (6)	−0.0061 (6)	−0.0054 (6)
N1	0.0481 (7)	0.0460 (7)	0.0435 (7)	0.0016 (5)	−0.0050 (6)	−0.0001 (6)
C1	0.0591 (10)	0.0511 (9)	0.0523 (9)	0.0110 (7)	0.0047 (8)	0.0065 (7)
C2	0.0691 (10)	0.0415 (8)	0.0684 (11)	0.0006 (7)	0.0041 (9)	0.0038 (8)
C3	0.0612 (10)	0.0449 (8)	0.0580 (10)	−0.0016 (7)	−0.0066 (8)	−0.0057 (7)
C4	0.0490 (8)	0.0436 (8)	0.0448 (8)	0.0055 (6)	−0.0029 (7)	−0.0017 (6)
C5	0.0430 (7)	0.0412 (7)	0.0456 (8)	0.0057 (6)	0.0019 (6)	−0.0023 (6)
C6	0.0502 (9)	0.0533 (9)	0.0457 (9)	0.0052 (7)	−0.0014 (7)	0.0000 (7)
C7	0.0501 (9)	0.0538 (9)	0.0436 (9)	0.0020 (7)	−0.0060 (7)	−0.0022 (7)
C8	0.0449 (8)	0.0508 (8)	0.0487 (9)	−0.0029 (7)	−0.0041 (7)	−0.0059 (7)
C9	0.0426 (8)	0.0482 (8)	0.0529 (9)	−0.0017 (6)	−0.0063 (7)	−0.0075 (7)
C10	0.0517 (9)	0.0491 (8)	0.0569 (10)	−0.0032 (7)	−0.0069 (7)	−0.0011 (7)
C11	0.0484 (9)	0.0501 (9)	0.0650 (11)	−0.0082 (7)	0.0013 (8)	−0.0106 (8)
C12	0.0581 (10)	0.0642 (11)	0.0682 (11)	−0.0130 (8)	−0.0182 (9)	−0.0096 (9)
C13	0.0612 (10)	0.0634 (10)	0.0576 (10)	−0.0062 (8)	−0.0178 (8)	−0.0039 (8)
C14	0.0588 (10)	0.0554 (9)	0.0659 (11)	−0.0070 (8)	0.0075 (9)	−0.0090 (8)
C15	0.0650 (11)	0.0543 (9)	0.0630 (11)	−0.0093 (8)	0.0210 (9)	−0.0150 (8)
C16	0.1016 (15)	0.0646 (12)	0.0650 (12)	−0.0107 (11)	0.0122 (11)	−0.0092 (10)
C17	0.140 (2)	0.0627 (12)	0.0685 (14)	−0.0114 (13)	0.0257 (14)	−0.0049 (10)
C18	0.127 (2)	0.0675 (13)	0.0879 (17)	−0.0338 (13)	0.0433 (15)	−0.0139 (12)
C19	0.0841 (15)	0.0798 (15)	0.1170 (19)	−0.0316 (12)	0.0161 (14)	−0.0185 (14)
C20	0.0707 (12)	0.0616 (11)	0.1026 (16)	−0.0153 (9)	0.0123 (11)	−0.0092 (10)
C21	0.0904 (14)	0.0737 (12)	0.0710 (13)	0.0068 (10)	−0.0014 (11)	0.0239 (10)

### Geometric parameters (Å, º)

**Table:**

O1—C4	1.357 (2)	C15—C20	1.379 (3)
O2—C9	1.287 (2)	C15—C16	1.384 (3)
O3—C11	1.365 (2)	C16—C17	1.382 (3)
O3—C14	1.419 (2)	C17—C18	1.366 (4)
O1—H1o	0.96 (2)	C18—C19	1.364 (3)
N1—C5	1.413 (2)	C19—C20	1.396 (3)
N1—C7	1.310 (2)	C2—H2	0.9300
N1—H1n	0.940 (19)	C3—H3	0.9300
C1—C6	1.385 (2)	C6—H6	0.9300
C1—C21	1.508 (3)	C7—H7	0.971 (17)
C1—C2	1.380 (3)	C10—H10	0.9300
C2—C3	1.378 (2)	C12—H12	0.9300
C3—C4	1.387 (2)	C13—H13	0.9300
C4—C5	1.393 (2)	C14—H14A	0.9700
C5—C6	1.385 (2)	C14—H14B	0.9700
C7—C8	1.396 (2)	C16—H16	0.9300
C8—C13	1.421 (2)	C17—H17	0.9300
C8—C9	1.433 (2)	C18—H18	0.9300
C9—C10	1.416 (2)	C19—H19	0.9300
C10—C11	1.369 (2)	C20—H20	0.9300
C11—C12	1.408 (3)	C21—H21A	0.9600
C12—C13	1.344 (2)	C21—H21B	0.9600
C14—C15	1.497 (3)	C21—H21C	0.9600
			
C11—O3—C14	117.87 (13)	C18—C19—C20	120.3 (2)
C4—O1—H1o	111.1 (13)	C15—C20—C19	120.28 (19)
C5—N1—C7	127.71 (13)	C1—C2—H2	119.00
C5—N1—H1n	119.9 (11)	C3—C2—H2	119.00
C7—N1—H1n	112.4 (11)	C2—C3—H3	120.00
C2—C1—C6	117.40 (15)	C4—C3—H3	120.00
C2—C1—C21	121.94 (15)	C1—C6—H6	119.00
C6—C1—C21	120.67 (15)	C5—C6—H6	119.00
C1—C2—C3	122.37 (15)	N1—C7—H7	116.6 (10)
C2—C3—C4	119.94 (15)	C8—C7—H7	118.9 (10)
C3—C4—C5	118.58 (14)	C9—C10—H10	120.00
O1—C4—C3	124.00 (14)	C11—C10—H10	120.00
O1—C4—C5	117.41 (13)	C11—C12—H12	120.00
N1—C5—C4	116.40 (13)	C13—C12—H12	120.00
N1—C5—C6	123.29 (13)	C8—C13—H13	119.00
C4—C5—C6	120.31 (13)	C12—C13—H13	119.00
C1—C6—C5	121.40 (14)	O3—C14—H14A	110.00
N1—C7—C8	124.48 (15)	O3—C14—H14B	110.00
C9—C8—C13	118.89 (14)	C15—C14—H14A	110.00
C7—C8—C9	121.29 (14)	C15—C14—H14B	110.00
C7—C8—C13	119.80 (14)	H14A—C14—H14B	108.00
O2—C9—C8	120.49 (13)	C15—C16—H16	120.00
O2—C9—C10	121.57 (14)	C17—C16—H16	120.00
C8—C9—C10	117.93 (14)	C16—C17—H17	120.00
C9—C10—C11	120.54 (15)	C18—C17—H17	120.00
C10—C11—C12	121.43 (15)	C17—C18—H18	120.00
O3—C11—C12	114.26 (14)	C19—C18—H18	120.00
O3—C11—C10	124.31 (15)	C18—C19—H19	120.00
C11—C12—C13	119.45 (16)	C20—C19—H19	120.00
C8—C13—C12	121.72 (16)	C15—C20—H20	120.00
O3—C14—C15	110.09 (14)	C19—C20—H20	120.00
C14—C15—C16	119.02 (16)	C1—C21—H21A	109.00
C14—C15—C20	122.43 (16)	C1—C21—H21B	109.00
C16—C15—C20	118.55 (17)	C1—C21—H21C	109.00
C15—C16—C17	120.6 (2)	H21A—C21—H21B	109.00
C16—C17—C18	120.52 (19)	H21A—C21—H21C	109.00
C17—C18—C19	119.73 (19)	H21B—C21—H21C	109.00
			
C14—O3—C11—C10	−3.0 (2)	C7—C8—C9—C10	−176.46 (14)
C14—O3—C11—C12	177.39 (14)	C13—C8—C9—O2	−178.95 (14)
C11—O3—C14—C15	179.43 (14)	C13—C8—C9—C10	1.8 (2)
C7—N1—C5—C4	−171.50 (15)	C7—C8—C13—C12	177.89 (16)
C7—N1—C5—C6	8.9 (2)	C9—C8—C13—C12	−0.4 (2)
C5—N1—C7—C8	179.88 (14)	O2—C9—C10—C11	178.81 (15)
C6—C1—C2—C3	−0.4 (3)	C8—C9—C10—C11	−1.9 (2)
C21—C1—C2—C3	179.41 (16)	C9—C10—C11—O3	−178.96 (14)
C2—C1—C6—C5	0.0 (2)	C9—C10—C11—C12	0.7 (2)
C21—C1—C6—C5	−179.84 (15)	O3—C11—C12—C13	−179.54 (15)
C1—C2—C3—C4	0.3 (3)	C10—C11—C12—C13	0.8 (3)
C2—C3—C4—O1	179.74 (15)	C11—C12—C13—C8	−0.9 (3)
C2—C3—C4—C5	0.3 (2)	O3—C14—C15—C16	−163.21 (16)
O1—C4—C5—N1	0.2 (2)	O3—C14—C15—C20	17.1 (2)
O1—C4—C5—C6	179.81 (14)	C14—C15—C16—C17	−178.97 (18)
C3—C4—C5—N1	179.66 (14)	C20—C15—C16—C17	0.7 (3)
C3—C4—C5—C6	−0.7 (2)	C14—C15—C20—C19	179.30 (19)
N1—C5—C6—C1	−179.83 (14)	C16—C15—C20—C19	−0.4 (3)
C4—C5—C6—C1	0.6 (2)	C15—C16—C17—C18	−0.9 (3)
N1—C7—C8—C9	−0.4 (2)	C16—C17—C18—C19	0.7 (4)
N1—C7—C8—C13	−178.58 (15)	C17—C18—C19—C20	−0.3 (4)
C7—C8—C9—O2	2.8 (2)	C18—C19—C20—C15	0.2 (4)

### Hydrogen-bond geometry (Å, º)

**Table:**

*D*—H···*A*	*D*—H	H···*A*	*D*···*A*	*D*—H···*A*
N1—H1*n*···O2	0.94 (2)	1.83 (2)	2.609 (2)	138.7 (16)
O1—H1*o*···O2^i^	0.96 (2)	1.63 (2)	2.590 (2)	176.1 (17)

Symmetry code: (i) −*x*, *y*−1/2, −*z*+1/2.
