# Supplementary figures and images for: Crystal structure of (Z)-3-benz­yloxy-6-[(2-hy­droxy-5-methyl­anilino)methyl­idene]cyclo­hexa-2,4-dien-1-one
Source: Acta Crystallogr Sect E Struct Rep Online. 2014 Aug 1;70(Pt 9):o957–8. doi: 10.1107/S1600536814016936 (PMC4186113; doi:10.1107/S1600536814016936)

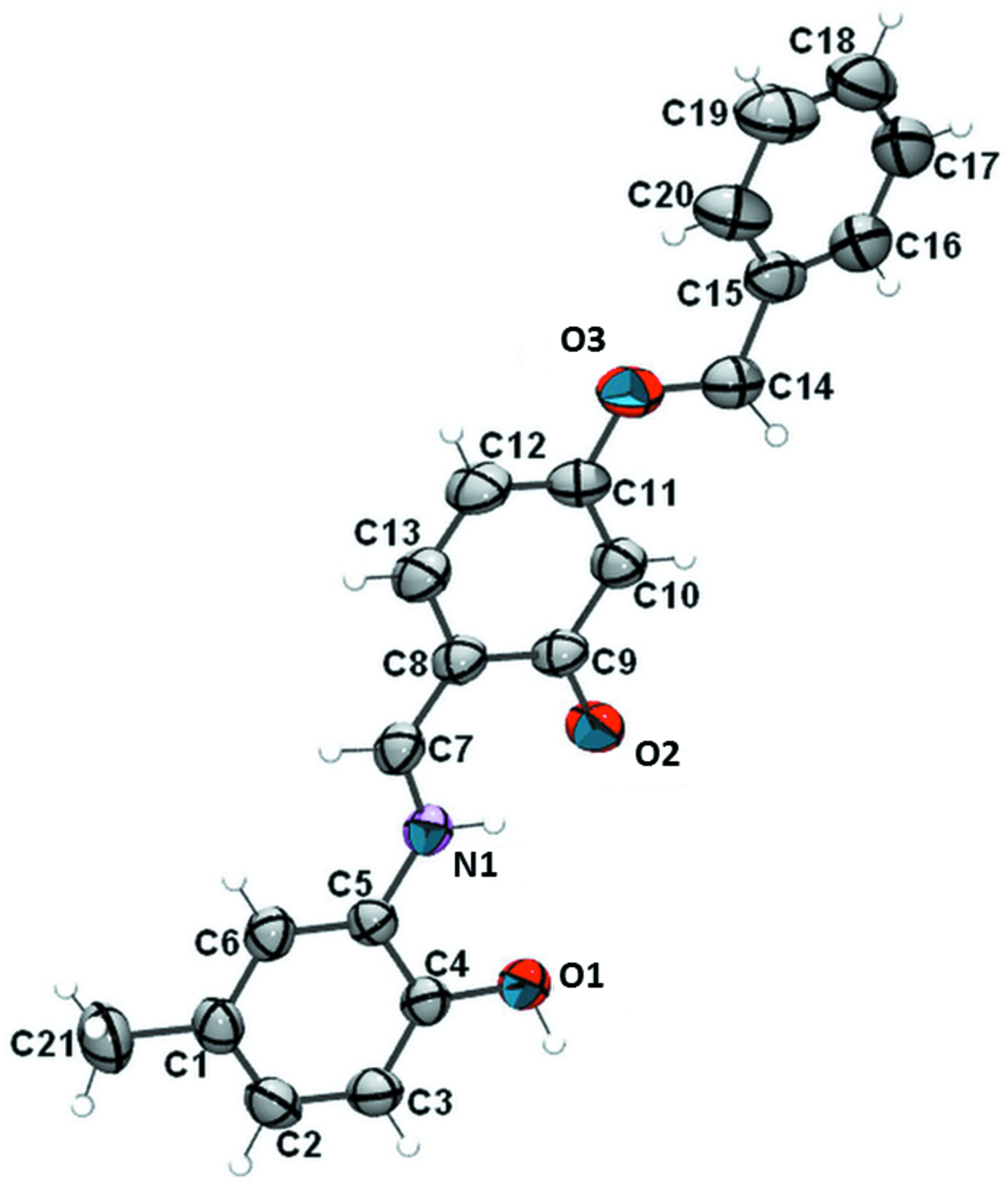

Supplement: Supplementary file 4 [file e-70-0o957-fig1.tif]

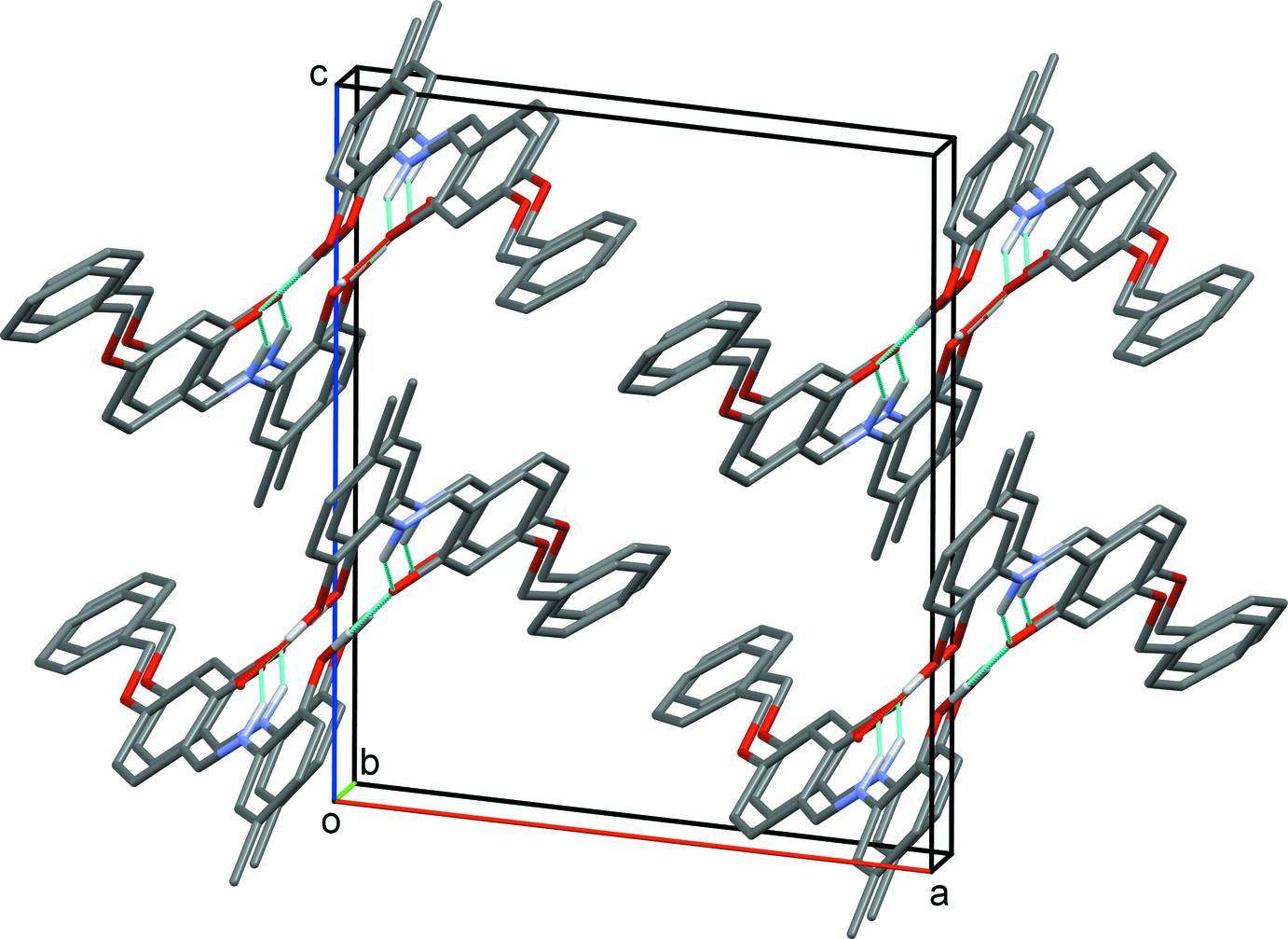

Supplement: Supplementary file 5 [file e-70-0o957-fig2.tif]
